# Community pharmacy transformation under universal health coverage: Implementation barriers, cultural determinants, and cross-national lessons from Japan's ongoing transition – A narrative review

**DOI:** 10.1016/j.rcsop.2026.100850

**Published:** 2026-09-20

**Authors:** Ryuta Urakawa, Kazumi Kubota, Risa Marikawa Urakawa, Rika Tanaka

**Affiliations:** a Department of Pharmacy, The University of Osaka Dental Hospital, 1-8 Yamada-oka, Suita, Osaka 565-0871, Japan; b Department of Clinical Pharmacy Research and Education, Graduate School of Pharmaceutical Sciences, The University of Osaka, 1-6 Yamada-oka, Suita, Osaka 565-0871, Japan; c Shimonoseki City University, 2-1-1 Daigaku-cho, Shimonoseki, Yamaguchi 751-8510, Japan; d Department of Healthcare Information Management, The University of Tokyo Hospital, 7-3-1 Hongo, Bunkyo-ku, Tokyo 113-8655, Japan; e National Center for Child Health and Development, 2-10-1 Okura, Setagaya-ku, Tokyo 157-8535, Japan; f Sugi Pharmacy Co., Ltd., 62-1 Shinye, Yokone-cho, Obu, Aichi 474-0011, Japan

**Keywords:** Implementation science, CFIR, Japan, Pharmacist services, Health policy, Cultural factors, International comparison

## Abstract

**Background:**

Community pharmacies worldwide are shifting from product-centered dispensing toward patient-centered clinical services. Japan achieved a prescribing–dispensing separation rate exceeding 80%, yet faces persistent challenges in delivering patient-centered services. This review examines why Japan's transformation has progressed slowly despite favorable structural conditions, and what lessons it offers other universal health coverage (UHC) systems.

**Methods:**

We conducted a narrative review using PubMed/MEDLINE and Ichushi-Web, covering literature primarily from 2016 to 2026. Six targeted searches addressed Japan's context, three tiers of comparator countries, and implementation science frameworks. From 2388 unique records, 87 publications were included. The updated Consolidated Framework for Implementation Research (CFIR 2.0) served as the analytical framework.

**Results:**

Tier 1 countries demonstrated that successful transformation requires aligned policy incentives, expanded scopes of practice, and robust outcome evidence. Tier 2 countries revealed shared cultural barriers, including physician-dominant hierarchies and dispensing-centered patient expectations. Japan's barriers spanned all five CFIR 2.0 domains: misaligned reimbursement, workforce shortages and absent pharmacy technicians, low pharmacist assertiveness, high service complexity, and fragmented implementation. Cultural factors—particularly high uncertainty avoidance and collectivism—interacted with institutional structures. Japan's family pharmacist system was associated with emerging evidence of benefit, including approximately 9% lower mortality risk, but limited adoption.

**Conclusions:**

Structural separation alone is insufficient without concurrent investment in workforce capability, practice authority, payment reform, and cultural adaptation. CFIR 2.0 effectively captures the multi-level nature of implementation barriers, supporting performance-based remuneration, scope expansion, and culturally sensitive strategies.

## Highlights

• Structural dispensing separation alone cannot ensure patient-centered care.
• CFIR 2.0 maps community pharmacy reform barriers across five domains.
• Culture interacts with institutions to slow Japan's pharmacy transformation.
• Japan's family pharmacist system links to ∼9% lower mortality risk.
• Payment reform and pharmacy technicians are key cross-national lessons.

## Background

Universal health coverage systems face a common paradox in community pharmacy reform: the infrastructure for dispensing is well established, yet the transition to patient-centered clinical services remains incomplete in many countries. This gap between structural capacity and functional transformation represents one of the most persistent challenges in primary healthcare reform globally.^1^, 2.

Community pharmacists are the most accessible healthcare professionals in most health systems, with the potential to contribute substantially to chronic disease management, medication optimization, preventive care, and public health delivery.^3^ The COVID-19 pandemic accelerated recognition of this potential, as pharmacies worldwide rapidly adapted to provide vaccination, screening, and emergency medication supply services.^4^ A review of the pandemic's impact on pharmacy practice documented expanded pharmacist roles across numerous countries, yet also noted that many of these expansions remained temporary or unevenly implemented.^4^

Japan presents an instructive case for understanding the barriers to community pharmacy transformation within UHC systems. Following decades of deliberate policy intervention, Japan achieved a prescribing–dispensing separation rate exceeding 80%, structurally positioning community pharmacies as independent healthcare providers rather than extensions of physician clinics.^5^, 6., 7 The 2015 “Pharmacy Vision for Patients” articulated a national policy direction toward patient-centered pharmaceutical services,^8^ subsequently reinforced by the 2019 amendment to the Pharmaceutical and Medical Device Act, which established legal categories for “community collaborative pharmacies” and “specialized pharmacies”.^9^ Despite these structural and legislative foundations, the actual delivery of patient-centered services has not yet reached its intended scale. National surveys reveal that medication follow-up consultations are conducted for only 0.84% of eligible patients,^10^ pharmacist-led deprescribing occurs at a rate of 0.08%,^11^ and public awareness of the Health Support Pharmacy designation remains at 12.2%.^12^

This implementation gap is not unique to Japan. Across 16 European nations, pharmaceutical care provision showed slow and uneven progress despite decades of advocacy.^13^ A comparative survey of 12 countries demonstrated varying levels of clinical pharmacy service development, with considerable disparity between educational preparation and actual practice delivery.^3^ These global patterns suggest that the barriers to community pharmacy transformation are systemic rather than unique to individual countries, yet the specific configurations of institutional, cultural, and individual factors differ significantly across national contexts.

The World Health Organization's health system building blocks framework provides a structural lens for understanding how financing, workforce, governance, and service delivery interact within UHC systems,^1^ while Hofstede's cultural dimensions theory offers a complementary perspective on how national cultural characteristics—including power distance, uncertainty avoidance, individualism–collectivism, and long-term orientation—shape healthcare performance and professional behavior.^14^, ^15^ A 35-country analysis demonstrated significant associations between cultural dimensions and health system outcomes, suggesting that culture may function as more than a contextual backdrop and could serve as a meaningful factor in implementation success.^15^

This review addresses three interconnected questions. First, why has Japan struggled to achieve meaningful patient-centered pharmacy practice despite having achieved structural prescribing–dispensing separation? Second, what institutional and cultural factors distinguish successful community pharmacy transformations in comparator countries? Third, what cross-national lessons can inform implementation strategies for Japan and other UHC systems facing similar challenges? To address these questions, this review employs the updated Consolidated Framework for Implementation Research (CFIR 2.0)^16^ as an analytical framework capable of capturing the multi-level, culturally embedded nature of implementation determinants. (Fig. 1).

**Fig. 1:**
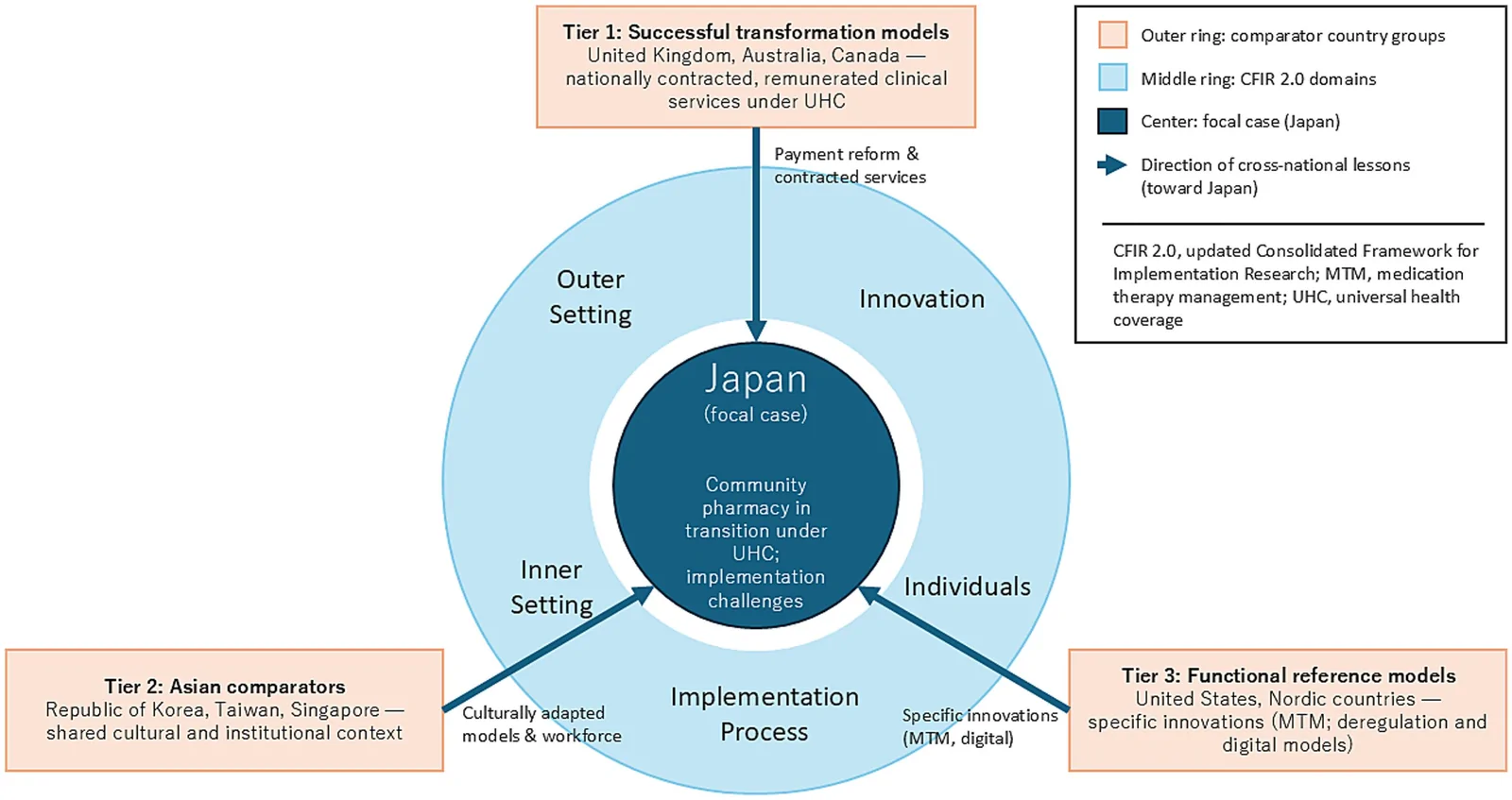
Conceptual framework for cross-national analysis of community pharmacy transformation using CFIR 2.0. Japan is positioned at the center as the focal country, encircled by the five domains of the updated Consolidated Framework for Implementation Research (CFIR 2.0)—Outer Setting, Inner Setting, Individuals, Innovation, and Implementation Process—through which its implementation challenges were analyzed. Three tiers of comparator countries are arranged in the outer ring: Tier 1 (United Kingdom, Australia, Canada), Tier 2 (Republic of Korea, Taiwan, Singapore), and Tier 3 (United States, Nordic countries), selected respectively as contracted clinical-service models, culturally and institutionally comparable Asian systems, and functional reference points for specific innovations. Inward-pointing arrows and their keyword annotations summarize the primary lessons each tier contributes to the analysis of Japan's transition; the arrows denote the overall direction of cross-national learning toward Japan and are not intended to map individual tiers onto specific CFIR 2.0 domains. The tiered structure is a heuristic analytical device rather than a ranking of system performance. CFIR 2.0, updated Consolidated Framework for Implementation Research; MTM, medication therapy management; UHC, universal health coverage.

## Methods

This study employed a narrative review methodology, following established guidance and methodological rationale for narrative-style literature reviews.^17^, 18. We deliberately chose a narrative rather than a systematic or scoping review because the research questions required interpretive synthesis across diverse evidence types—including policy analyses, implementation studies, qualitative research, randomized controlled trials, and grey literature—spanning multiple countries with distinct healthcare systems and cultural contexts. This breadth and heterogeneity of evidence precluded the protocol-driven aggregation and meta-analysis that characterize systematic reviews, as well as the evidence-mapping aims of scoping reviews, while the interpretive, theory-driven nature of our inquiry aligned with the strengths of narrative synthesis.^19^ The structured search documentation and the adapted flow diagram (Fig. 2) are provided solely to enhance transparency and reproducibility and do not imply adherence to systematic review methodology.

**Fig. 2:**
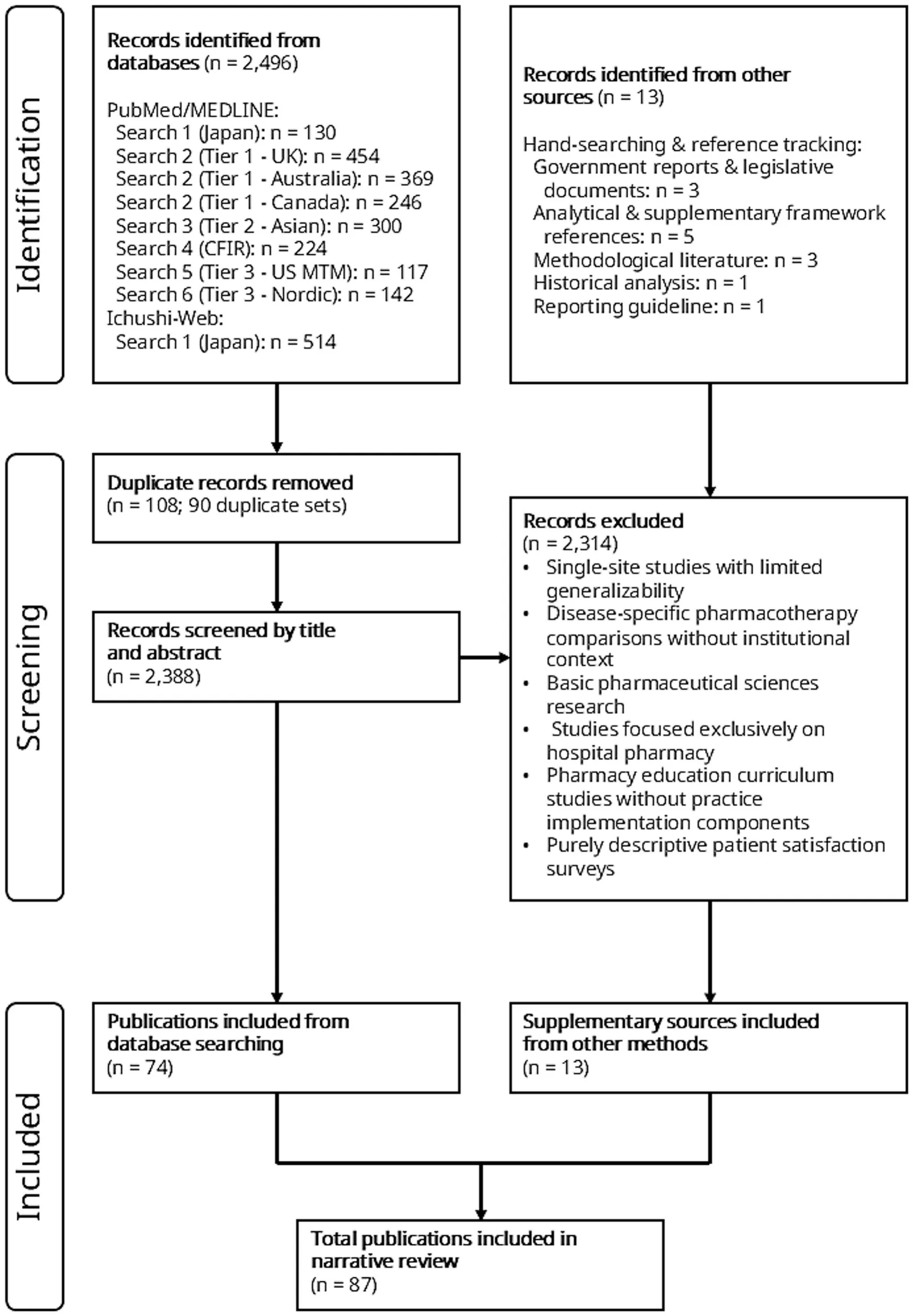
Flow diagram of the literature selection process, adapted from the PRISMA 2020 template. Note: This is a narrative review. The diagram is adapted from the PRISMA 2020 template^22^ for transparency of the literature selection process only, and does not indicate adherence to PRISMA or compliance with systematic or scoping review methodology. Adapted from: Page MJ, et al. The PRISMA 2020 statement: an updated guideline for reporting systematic reviews. BMJ. 2021;372:n71.^22^ Screening was conducted by a single reviewer (RU). Supplementary sources were identified through hand-searching and reference tracking of government reports, framework documents, methodological literature, and a reporting guideline essential to the analysis. CFIR, Consolidated Framework for Implementation Research; MTM, medication therapy management; UK, United Kingdom; US, United States.

Literature searches were conducted across two electronic databases: PubMed/MEDLINE and Ichushi-Web (Japan Medical Abstracts Society) (Supplementary File 1). Six separate searches were performed targeting: (1) Japan's community pharmacy context; (2) Tier 1 comparator countries (United Kingdom, Australia, and Canada) with established community pharmacy clinical service frameworks under UHC; (3) Tier 2 comparator countries (Republic of Korea, Taiwan, and Singapore) sharing cultural and institutional similarities with Japan; (4) studies applying the Consolidated Framework for Implementation Research (CFIR) to pharmacy settings; (5) United States medication therapy management implementation; and (6) Nordic countries' integrated care and digital pharmacy models. Search strategies combined Medical Subject Headings terms and free-text keywords related to community pharmacy services, pharmacist roles, pharmaceutical care, and country-specific terms. For most searches, the period covered January 2016 to February 2026. The start date of January 2016 was chosen as the year following Japan's 2015 “Pharmacy Vision for Patients,” to capture the most recent decade of reform literature most directly relevant to the current policy context. For the CFIR, Asian, and Nordic searches, the period was extended back to January 2011 because the foundational literature in these areas emerged earlier: CFIR–pharmacy applications began around 2013, key Asian prescribing–dispensing separation studies were published in the early 2010s, and Nordic deregulation literature included important pre-2016 analyses. The Japan and Tier 1 searches did not require this extension, as their most policy-relevant literature is concentrated within the post-2016 period. Searches were limited to English- and Japanese-language publications. The non-inclusion of Embase is acknowledged as a limitation. The complete search strings, Boolean operators, database-specific strategies, and record counts for each of the six searches are provided in Supplementary File 1 to support transparency and reproducibility.

The initial search yielded 2496 records. After removal of 108 duplicates (90 duplicate sets), 2388 unique records underwent title and abstract screening. Inclusion criteria prioritized reviews and policy analyses providing comprehensive overviews of national community pharmacy systems, studies applying CFIR or other implementation science frameworks to pharmacy practice, research directly addressing Japan's community pharmacy reforms, multi-country comparative studies, and evaluations of key institutional innovations. Exclusion criteria comprised single-site small-scale studies with limited generalizability, disease-specific pharmacotherapy comparisons without institutional context, basic pharmacological research, studies focused exclusively on hospital pharmacists, pharmacy education research, and purely descriptive patient satisfaction surveys. An evidence-driven depth approach was adopted, whereby the breadth and quality of available literature for each country naturally determined the level of analytical detail. From 2388 unique records, 74 publications were selected through title and abstract screening based on predefined inclusion and exclusion criteria. Thirteen additional supplementary sources were identified through hand-searching and reference tracking: these comprised government reports and legislative research documents essential to the analysis of Japan's pharmacy reform trajectory,6., 7, 8 the primary analytical and supplementary framework references 1, 2., 14, 16, 20, methodological literature supporting the narrative review design,^17^, 18., 19 a historical analysis of the Japanese pharmacist's role during the COVID-19 pandemic,^21^ and a reporting guideline adopted to enhance transparency of the literature selection process.^22^ In total, 87 publications were included in the analysis. While this study employs a narrative review methodology, a flow diagram adapted from the PRISMA 2020 template^22^ is provided to enhance transparency of the literature selection process (Fig. 2).

The updated CFIR (CFIR 2.0)^16^ served as the primary analytical framework, supplemented by the CFIR Outcomes Addendum.^20^ CFIR 2.0 was selected for its comprehensive coverage of implementation determinants across five domains—Innovation, Outer Setting, Inner Setting, Individuals, and Implementation Process—and its explicit accommodation of cultural and contextual factors through constructs such as Local Attitudes, Critical Incidents, and Recipient-Centeredness. The adaptation of the original CFIR to community pharmacy settings has been previously validated,^23^ supporting the applicability of its updated version to this context. The WHO Health System Building Blocks framework^1^ was used for background contextualization, and Hofstede's cultural dimensions theory^14^ informed cross-national cultural analysis.

Selected literature was organized by mapping findings to CFIR 2.0 domains, with comparative analysis structured across the three-tier country framework. Mapping of findings to CFIR 2.0 constructs was conducted by the lead author (RU) through iterative close reading of each included source, with each relevant finding assigned to the most salient domain and construct. To reduce interpretive bias, provisional mappings were reviewed and discussed with the co-authors (KK, RMU, RT); disagreements were resolved through discussion until consensus was reached, and ambiguous cases were assigned to the domain judged most consistent with the CFIR 2.0 construct definitions. As in any narrative synthesis, this mapping involved interpretive judgment; the resulting structure is intended as an analytical lens rather than a validated coding scheme.

The three-tier structure was defined by analytical purpose rather than by a ranking of overall system performance. Tier 1 (United Kingdom, Australia, Canada) comprised UHC systems with nationally contracted, remunerated community pharmacy clinical services and a substantial peer-reviewed implementation literature, selected as models from which transferable lessons could be drawn. Tier 2 (Republic of Korea, Taiwan, Singapore) comprised Asian UHC systems sharing cultural and institutional features with Japan, selected to control partially for cultural context. Tier 3 (United States, Nordic countries) comprised systems included as functional reference points for specific innovations (medication therapy management; market deregulation and digital models) rather than as comprehensive transformation models. Other UHC systems with notable pharmacy reforms, such as the Netherlands, Germany, France, and New Zealand, could offer additional lessons; they were not included as primary comparators in order to keep the comparison analytically tractable and to prioritize systems either sharing Japan's cultural and institutional context or offering the most extensively documented contractual clinical-service models. The tiered structure is therefore a heuristic analytical device rather than a rigid or exhaustive classification. The label “successful transformation models” for Tier 1 denotes relative progress in establishing remunerated clinical services, not the absence of implementation barriers; as documented in this review, tensions and unresolved challenges persist even in these systems. Supplementary sources, including framework documents and theoretical works, were identified through reference tracking where they provided essential contextual information.

## International comparative analysis

### Analytical foundation

Before examining individual countries, it is important to note that systematic reviews using CFIR to analyze community pharmacy implementation have identified recurring patterns across national contexts. A review of national-level pharmacy innovations found that Outer Setting factors—particularly policy frameworks and financing mechanisms—were the most frequently cited determinants of implementation success or failure.^24^ Analysis of outpatient pharmaceutical care implementation confirmed that available resources, knowledge and beliefs of individuals, and external policy and incentives constituted the most prominent barriers and facilitators across settings.^25^ Furthermore, the complexity of clinical pharmacy interventions relative to traditional dispensing has been shown to interact with multiple implementation domains, suggesting that simplification and adaptation strategies are essential components of successful transformation.^26^ (Table 1).

**Table 1:** Comparative overview of community pharmacy systems.

Country	UHC model	PD separation	Pharmacist prescribing	Key clinical services	Payment for clinical services	Pharmacy technician role
UK	NHS (tax)	Full	Independent (expanded)	Pharmacy First, NMS, vaccination, PQS	FFS + P4P	Registered (GPhC)
Australia	Tax + private	Partial	None ^a^	MedsCheck, HMR, vaccination	CPA-based FFS	State-regulated
Canada	Provincial single-payer	Full	Provincial variation ^b^	Med review, minor ailments, vaccination	Provincial FFS/P4P	Provincial-regulated
Korea	NHI (social insurance)	Mandatory (2000)	None	Limited counseling	FFS (dispensing)	Non-licensed assistants
Taiwan	NHI (single-payer)	Partial ^c^	None	PharmaCloud, chronic Rx dispensing, home visits	NHI FFS	Pharmacy assistants
Singapore	Mixed ^d^	No formal separation	Collaborative (limited) ^e^	Physician-led model; counseling	FFS (dispensing)	Pharmacy technicians
US	No UHC ^f^	Full	Collaborative practice agreement (state-variable) ^g^	MTM, vaccination, point-of-care testing	Part D MTM; limited FFS	Certified/registered
Sweden/Finland	Tax (universal)	Full	Limited	Med review, e-prescription, self-care	FFS (dispensing); limited	Two pharmacist tiers ^h^; technicians
Japan	NHI (social insurance)	Mandatory (>80%)	None	Family pharmacist, HSP, med follow-up	FFS (dispensing dominant)	None

Abbreviations: CPA, Community Pharmacy Agreement; FFS, fee-for-service; GPhC, General Pharmaceutical Council; HMR, Home Medicines Review; HSP, Health Support Pharmacy; MTM, medication therapy management; NHI, National Health Insurance; NHS, National Health Service; NMS, New Medicine Service; P4P, pay-for-performance; PD, prescribing–dispensing; PQS, Pharmacy Quality Scheme; Rx, prescription; UHC, universal health coverage.

Notes: ^a^ Under discussion at state/territory level. ^b^ Alberta: additional prescribing authority; most provinces: minor ailments, renewals, adaptations. ^c^ Hospital outpatient dispensing remains common. ^d^ Medisave/Medishield/Medifund. ^e^ Limited to pharmacists completing the Ministry of Health collaborative prescribing programme. ^f^ Medicare/Medicaid provide partial coverage. ^g^ No federal provider status under Medicare Part B. ^h^ Both Sweden and Finland have two licensed pharmacist categories with independent dispensing authority: a 5-year Master's level (Sweden: apotekare; Finland: proviisori) and a 3-year Bachelor's level (Sweden: receptarie; Finland: farmaseutti). In both countries, pharmacy technicians (Sweden: apotekstekniker; Finland: lääketyöntekijä) provide logistical support. These distinct roles should not be conflated. This table provides a high-level overview; substantial within-country variation—particularly across Canadian provinces, Australian states and territories, US states, and the individual Nordic countries—is not fully captured here and is described in the main text.

### Tier 1: Successful transformation models

#### United Kingdom

The United Kingdom's National Health Service provides one of the most extensively documented models of community pharmacy clinical service integration within a UHC system. The NHS community pharmacy contractual framework has evolved through successive five-year agreements, progressively shifting remuneration from dispensing volume toward clinical service delivery.^27^ The 2024 Pharmacy First services represent the culmination of this trajectory, enabling community pharmacists to assess, treat, and supply medications for specified clinical conditions without prior physician consultation. To support safe and effective integration, a comprehensive CFIR-informed study protocol has been developed for evaluating the ongoing implementation and its health system impact.^28^ Independent prescribing by pharmacists has been progressively expanded, with a qualitative systematic review identifying organizational support, interprofessional relationships, and regulatory clarity as key facilitators for implementation in primary care settings.^29^ The New Medicine Service, evaluated through both a randomized controlled trial demonstrating approximately 10% improvement in medication adherence^30^ and a CFIR-based organizational ethnography revealing the practical challenges of workflow integration,^31^ illustrates how evidence generation and implementation analysis can proceed in parallel. The Pharmacy Quality Scheme introduced pay-for-performance elements, providing financial incentives for quality indicators rather than dispensing volume.^32^ However, research on pharmacist and general practitioner perspectives reveals persistent tensions regarding professional boundaries and collaborative relationships, with solidarity and dissonance coexisting within interprofessional dynamics.^33^ Organizational factors including pharmacy ownership type, skill mix configuration, and workplace culture have been shown to significantly influence clinical productivity, suggesting that structural reforms alone are insufficient without attention to organizational context.^34^

#### Australia

Australia's community pharmacy system operates through the Community Pharmacy Agreement (CPA), a five-yearly negotiated agreement between the government and the Pharmacy Guild of Australia that structures both remuneration and service delivery expectations.^35^ Policy analysis has characterized the CPA as occupying an inherent tension between industrial policy protecting pharmacy business viability and health policy promoting clinical service expansion.^36^ Within this framework, professional pharmacy services have been implemented through iterative cycles of development and evaluation. A CFIR-informed qualitative study applying the Expert Recommendations for Implementing Change (ERIC) taxonomy identified a six-stage implementation process for professional services, emphasizing the importance of tailored facilitation strategies.^37^ A semi-structured survey of pharmacists using CFIR constructs found that Inner Setting factors—particularly workforce availability and workflow infrastructure—were rated as the most critical implementation priorities, outweighing external policy factors in day-to-day practice.^38^ A systematic review of clinical medication review services in Australian community pharmacies documented positive impacts on medication-related problems but noted inconsistent implementation across practice settings.^39^ Multi-level analysis of factors influencing community pharmacy service delivery identified barriers operating simultaneously at micro (individual pharmacist), *meso* (organizational), and macro (system) levels, with policy-level factors shaping the boundaries of what was achievable at practice level.^40^

#### Canada

Canada's community pharmacy system is characterized by provincial jurisdiction over pharmacy regulation, creating what has been described as a “patchwork” of varying scopes of practice, reimbursement mechanisms, and service delivery models across the country.^41^ This decentralized structure has, paradoxically, enabled innovation by allowing individual provinces to serve as natural experiments in pharmacy reform. Alberta's compensation plan, developed through stakeholder consultation and implemented progressively, demonstrated that physician-equivalent remuneration for clinical services could drive a dramatic increase in service delivery, from approximately 30,000 to 170,000 monthly clinical claims—a 5.7-fold increase.^42^ Qualitative research with family physicians revealed that pharmacist prescribing was accepted when supported by transparent communication and established trust, though concerns about fragmentation of care persisted.^43^ The landmark RxEACH trial demonstrated that pharmacist-led cardiovascular risk management reduced estimated cardiovascular risk by 21% compared with usual care, providing robust evidence for pharmacist prescribing effectiveness.^44^ Systematic reviews of pharmacist prescribing roles and competencies across multiple countries identified training models, collaborative practice frameworks, and regulatory clarity as common enablers.^45^, ^46^ An international comparison of pharmacy payment models across OECD jurisdictions documented the shift from fee-for-service dispensing reimbursement toward performance-based and capitation models, with payment reform consistently identified as a necessary precondition for service transformation.^47^ A randomized controlled study of practice facilitation in community pharmacy demonstrated that external facilitation support significantly improved the adoption of clinical services, suggesting that implementation support infrastructure is as important as service design.^48^ (Table 2).

**Table 2:** CFIR 2.0 domain mapping across Tier 1 countries.

CFIR 2.0 domain	Construct	UK	Australia	Canada
Outer Setting	Financing	(+) Progressive shift to service-based contractual framework^27^	(+) CPA provides structured funding for professional services^35^; (−) Tension between industrial and health policy goals^36^	(+) Alberta compensation plan: physician-equivalent remuneration drove 5.7-fold increase in claims^42^
	Policies & Laws	(+) Pharmacy First services enable pharmacist assessment and treatment^28^; (+) Independent prescribing legislation^29^	(−) CPA negotiation cycle creates policy uncertainty^36^	(+) Provincial autonomy enables natural experiments; (−) “Patchwork” of inconsistent regulations^41^
	Local Attitudes	(−) GP–pharmacist tensions persist^33^	(−) Multi-level barriers at macro/*meso*/micro levels^40^	(+) Physician acceptance when trust established^43^; (−) Concerns about care fragmentation^43^
Inner Setting	Work Infrastructure	(+) Pharmacy technicians enable task redistribution^34^	(−) Workforce availability rated as top implementation priority^38^	(+) Practice facilitation improved clinical service adoption^48^
	Culture incl. Recipient-Centeredness	(−) Ownership type and workplace culture influence productivity^34^	(+) ERIC-based tailored facilitation strategies^37^	(+) Stakeholder consultation culture in policy development^42^
Individuals	Capability	(+) Independent prescribing training established^29^	(−) Inconsistent clinical skill application across settings^39^	(+) Robust evidence from RxEACH trial supports pharmacist competence^44^
	Motivation	(+) PQS financial incentives for quality^32^	(+) Professional aspiration for expanded roles^37^	(+) Financial incentives align with clinical activity^42^
Innovation	Evidence Base	(+) NMS RCT: ∼10% adherence improvement^30^	(+) Medication review reduces medication-related problems^39^	(+) RxEACH: 21% estimated CV risk reduction^44^
	Complexity	(−) NMS workflow integration challenges^31^	(−) Inconsistent implementation despite evidence^39^	(+/−) Prescribing roles require clear competency frameworks^45^, ^46^
Impl. Process	Reflecting & Evaluating	(+) CFIR-informed Pharmacy First evaluation protocol^28^	(+) CFIR/ERIC-informed iterative implementation^37^	(+) Practice facilitation RCT provides implementation evidence^48^
	Tailoring Strategies	(+) PQS adapted to local pharmacy contexts^32^	(+) Implementation priorities identified through CFIR survey^38^	(+) Payment model adapted through jurisdictional scanning^47^

Abbreviations: CPA, Community Pharmacy Agreement; CV, cardiovascular; ERIC, Expert Recommendations for Implementing Change; GP, general practitioner; Impl., Implementation; NMS, New Medicine Service; PQS, Pharmacy Quality Scheme; RCT, randomized controlled trial. (+) denotes facilitator; (−) denotes barrier; (+/−) denotes mixed evidence. Reference numbers correspond to the main text reference list. Each cell entry is an evidence-based finding drawn from the cited source; the assignment of findings to CFIR 2.0 domains and constructs, and the facilitator/barrier designations, reflect the authors' interpretive mapping (see Methods).

### Tier 2: Asian comparators with shared cultural contexts

#### Republic of Korea

Korea's experience with prescribing–dispensing separation provides a direct comparator to Japan, as both countries implemented separation policies within Asian healthcare systems characterized by physician-dominant hierarchies. Analysis of pharmaceutical consumption in areas exempt from Korea's separation mandate revealed that separation alone does not guarantee rational medication use, underscoring the need for complementary clinical services.^49^ A survey of public acceptance toward a “Family Pharmacy” model—analogous to Japan's “family pharmacist” system—found moderate acceptance but significant gaps in public understanding of expanded pharmacist roles.^50^ Public perception research confirmed that while Koreans increasingly acknowledged the importance of community pharmacies, awareness of clinical service capabilities remained limited, particularly for chronic disease management.^51^ The government's Prescribing Analysis System generated notable physician resistance, with qualitative research revealing tensions between regulatory oversight and perceived clinical autonomy.^52^ Good Pharmacy Practice implementation faced barriers primarily attributed to institutional factors rather than individual pharmacist resistance.^53^

#### Taiwan

Taiwan's single-payer National Health Insurance system has enabled system-wide digital integration that offers instructive lessons for pharmacy informatics. The PharmaCloud system, evaluated using the RE-AIM framework, demonstrated that cloud-based medication record sharing across all pharmacies and hospitals could improve medication safety and reduce duplication.^54^ Quantitative analysis confirmed significant reductions in medication duplication following PharmaCloud implementation.^55^ Strategic interventions to promote chronic disease prescription dispensing at community pharmacies, analyzed through interrupted time series methods, showed that policy incentives could gradually shift prescriptions from hospital outpatient departments to community settings, paralleling Japan's prescribing–dispensing separation trajectory.^56^ A pharmacist home visit project targeting high-frequency healthcare utilizers reduced physician visits, providing evidence for pharmacist-led patient management within the NHI framework.^57^ During the COVID-19 pandemic, Taiwan's community pharmacy network served as the primary infrastructure for real-name mask distribution, demonstrating the public health utility of an integrated pharmacy network connected through the NHI system.^58^

#### Singapore

Singapore's healthcare system adopts a physician-led, pharmacist-enabled approach, as reflected in the Healthier SG policy framework and recent specialist pharmacy position papers advocating structured pharmacist roles under physician oversight.^59^ Qualitative research with general practitioners revealed limited enthusiasm for expanded pharmacist clinical roles, with business competition and concerns about patient loyalty identified as primary barriers to collaboration.^60^ Even within hospital settings, patient and caregiver understanding of pharmaceutical care remained limited, with expectations centered on dispensing accuracy and product availability rather than clinical consultation.^61^

### Tier 3: Functional reference models

#### United States

Medication therapy management programs in the United States provide instructive examples of CFIR application to community pharmacy implementation. A CFIR-based evaluation of Tennessee's MTM pilot identified early-stage contextual factors influencing implementation, including organizational readiness, leadership engagement, and available resources.^62^ Analysis of barriers and facilitators for community pharmacist population health management highlighted the critical role of payment reform and provider status legislation in enabling expanded services.^63^ Notably, a Virginia statewide protocols study applying CFIR 2.0 specifically demonstrated the utility of the updated framework's constructs, including Local Attitudes and Recipient-Centeredness, for capturing the cultural dimensions of pharmacy practice transformation.^64^

#### Nordic countries

Sweden's experience following pharmacy market deregulation illustrates the tension between commercialization and clinical service development. The shift from a state pharmacy monopoly to a competitive market increased geographical access but raised concerns about whether commercial pressures would disincentivize time-intensive clinical services.^65^ Finland's policy roadmap for community pharmacy development, led by the pharmacy profession with government support, articulated a vision integrating digitalization, clinical service expansion, and regulatory modernization within a framework of regulated private pharmacy ownership.^66^

## Japan's context and implementation barriers

### Historical and policy context

Understanding Japan's current implementation challenges requires appreciating the historical trajectory of community pharmacy within the Japanese healthcare system. The 150-year history of Japanese pharmacy has been characterized by a gradual, contested evolution from a product-centered, physician-subordinate role toward an independent clinical profession.^5^, 6. The policy of prescribing–dispensing separation (iyaku bungyo), pursued through incremental reimbursement incentives over several decades, achieved its structural objective of establishing independent community pharmacies: the prescribing–dispensing separation rate rose from 11.3% in 1989 to 80.3% in 2023.^7^ Notably, reimbursement for patient-oriented services was first introduced in 1983 through the establishment of pharmacy management fees (yakugaku kanri-ryo),^6^ yet more than four decades later, the reimbursement structure continues to disproportionately reward dispensing volume over clinical service delivery.^67^ However, critical analyses have noted that the separation policy has not yet been accompanied by commensurate legal provisions for expanded pharmacist authority. It has been argued that recent policy revisions—such as changes to dispensing fees—may have prioritized healthcare cost containment over the legislative development needed to support broader professional autonomy.^67^

The 2019 amendment to the Pharmaceutical and Medical Device Act established legal definitions for community collaborative pharmacies (chiiki-renkei yakkyoku) and specialized pharmacies (senmon-iryo-kikan renkei yakkyoku), creating institutional categories for pharmacies providing enhanced clinical and collaborative services.^9^ These legislative changes, building on the 2015 Pharmacy Vision,^8^ articulated a clear national policy direction. Yet a substantial gap persists between legislative intent and practice implementation. (Fig. 3).

**Fig. 3:**
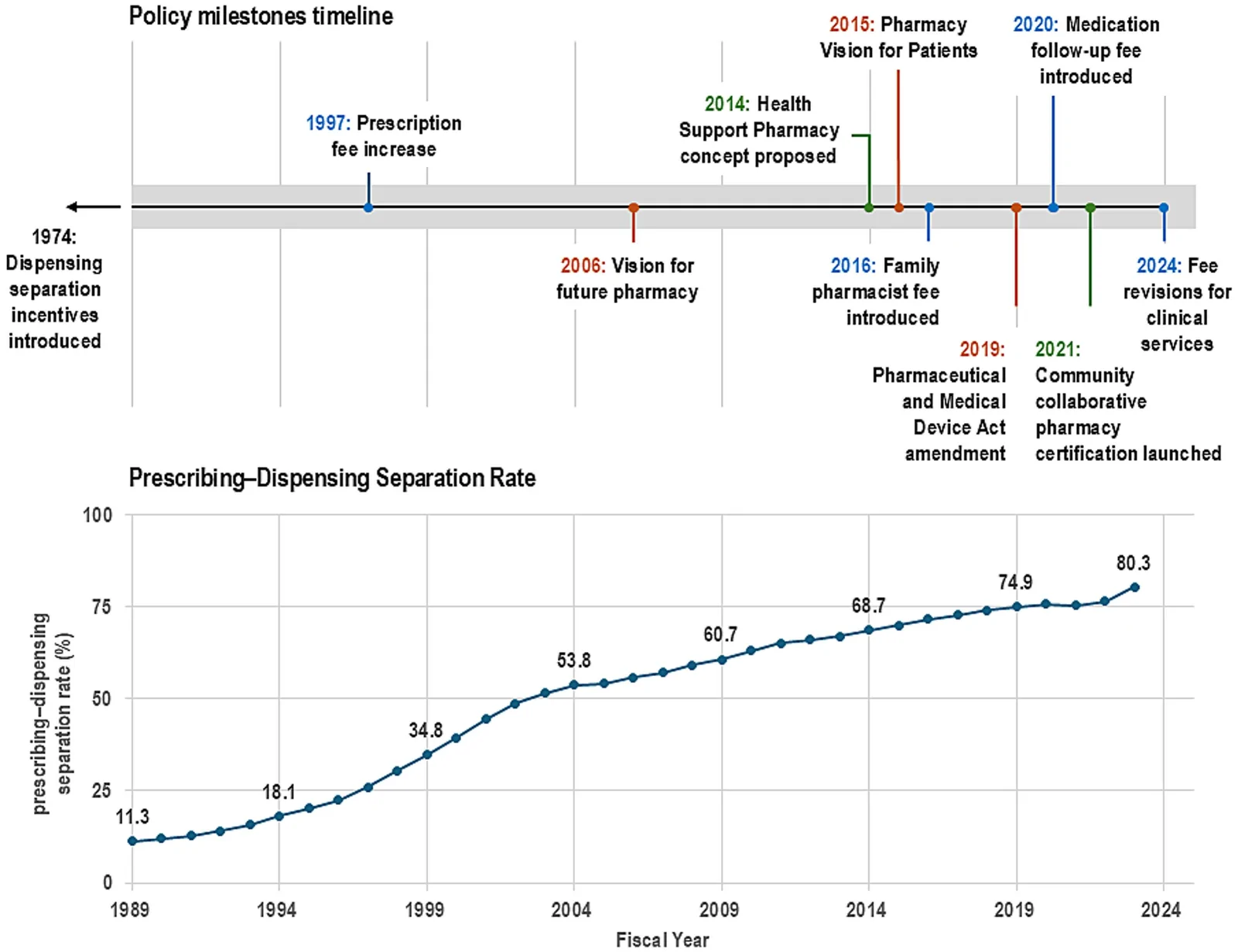
Timeline of Japan's community pharmacy policy evolution (1974–2024) and prescribing–dispensing separation rate progression. The upper panel displays key policy milestones categorized by type: reimbursement reforms (blue), legislation and national policy (red), and system/institutional milestones (green). The 1974 policy starting point is indicated by an off-axis annotation, as quantitative data are available from fiscal year 1989 onward. The lower panel plots the prescribing–dispensing separation rate from FY1989 (11.3%) to FY2023 (80.3%), based on Ministry of Health, Labour and Welfare data.^7^ The slight decline in FY2021 (75.3%) reflects the impact of the COVID-19 pandemic on outpatient prescription volumes. Policy milestones were compiled from legislative records^8^, ^9^ and published analyses.^5^, 67. This image was created with assistance of Claude (Anthropic). (For interpretation of the references to colour in this figure legend, the reader is referred to the web version of this article.)

### Outer Setting barriers: policy misalignment and cultural attitudes

Japan's reimbursement system exemplifies the Outer Setting construct of Financing within CFIR 2.0. Under the current fee schedule, the dispensing fee (chozai gijutsu-ryo) and the drug management and guidance fee (yakuzai kanri shido-ryo) form the remuneration core, while patient-oriented activities such as medication follow-up and deprescribing are compensated through comparatively small add-on fees rather than being integrated into the basic technical fees.^67^ Because the base fees remain tied largely to prescription volume, formulation complexity, and pharmacy location rather than to the clinical services actually provided, the marginal financial return on time-intensive clinical activities is low relative to high-throughput dispensing.^67^ This creates a structural disincentive for pharmacies to invest in clinical service capacity, particularly given the economic pressures facing community pharmacies operating in a competitive market. An international comparison of pharmacy payment models confirms that fee-for-service dispensing reimbursement consistently inhibits clinical service development across jurisdictions.^47^ Plausible reforms within the national health insurance framework include rebalancing the base fees toward patient-oriented services, converting selected clinical add-on fees into core technical fees to signal their essential nature, and introducing performance-linked components analogous to the UK Pharmacy Quality Scheme^32^; whether such reforms are politically and fiscally feasible within Japan's cost-containment environment remains an open question.

Cultural attitudes constitute a powerful and underexplored implementation determinant. A 35-country analysis of cultural dimensions and health system performance found that Japan scores notably high on uncertainty avoidance and mid-range individualism, lower than Western comparators but notably higher than other East Asian nations,^15^ characteristics that may be associated with risk-averse professional behavior and deference to hierarchical authority. In the pharmacy context, this manifests as reluctance among pharmacists to challenge physician prescribing decisions and low rates of proactive clinical intervention. A cross-sectional study of pharmacist assertiveness found significant associations between self-assertiveness scores and deprescribing experience, suggesting that individual behavioral characteristics mediate the relationship between structural opportunity and clinical action.^68^ Qualitative research using the Theory of Planned Behavior revealed that both physicians and pharmacists perceived deprescribing as professionally risky, with subjective norms favoring non-intervention over proactive medication optimization.^69^

### Inner Setting barriers: workforce and organizational constraints

Japan is notable among developed nations for the absence of a formally recognized pharmacy technician role. An international survey of pharmacy support workforces across 67 countries documented Japan's position as an outlier in this regard.^70^ This structural gap means that pharmacists must perform all dispensing and administrative tasks themselves, substantially limiting the time available for clinical services. National survey data confirm that workforce shortages and excessive dispensing workload are the most frequently cited barriers to expanded clinical service delivery.^71^

The organizational culture of Japanese community pharmacies, analyzed through the CFIR 2.0 construct of Recipient-Centeredness, reflects broader cultural norms of service orientation (omotenashi) that paradoxically may impede clinical assertiveness. A qualitative study using the Donabedian framework identified that Health Support Pharmacy service quality was influenced by structural factors (staffing, space, equipment), process factors (consultation skills, documentation), and outcome factors (patient satisfaction), with structural limitations fundamentally constraining process improvements.^72^

### Individuals: capability, opportunity, and motivation gaps

Analysis through the CFIR 2.0 Individuals domain, structured by COM-B (Capability, Opportunity, Motivation), reveals multilayered challenges. Application of the FIP Global Competency Framework to Japanese pharmacists identified significant gaps in organizational management and professional development competencies relative to international benchmarks.^73^ A comparative study of Japanese and Irish pharmacists' attitudes toward diabetes management found that Japanese pharmacists reported lower confidence and narrower self-perceived scope of practice.^74^

Research on pharmacist behavior provides further insight into the gap between capability and action. A qualitative investigation of barriers experienced by pharmacists who were motivated to propose deprescribing but faced obstacles in doing so, identified fear of damaging the physician–pharmacist relationship, time constraints, and lack of institutional support as primary barriers.^75^ These findings align with national survey data showing that even trained pharmacists deliver medication follow-up at rates below 1%^10^ and pharmacist-led deprescribing at 0.08% of eligible encounters.^11^

### Innovation characteristics: emerging evidence and adoption paradox

Despite these implementation challenges, emerging evidence indicates that Japan's pharmacy service innovations, when delivered, are associated with meaningful outcomes. A large-scale cohort study of the “family pharmacist” (kakaritsuke-yakuzaishi) system was associated with an approximately 9% lower mortality risk among patients with cardiovascular and endocrine conditions.^76^ The system was also associated with a lower incidence of therapeutic drug–drug interactions among underage individuals.^77^ Research examining factors associated with family pharmacist prescription modifications found that family pharmacists were 1.37 times more likely to achieve prescription changes compared with non-designated pharmacists.^78^ The “My Pharmacist/My Pharmacy” model demonstrated associations with improved service quality across multiple dimensions.^79^

Yet the gap between evidence and adoption is substantial. National survey data indicate that the family pharmacist consent rate stands at approximately 2.9%,^80^ Health Support Pharmacy awareness at 12.2%,^12^ and a broader patient awareness survey found that only 22.2% of respondents were aware of the family pharmacist system, though 40.7% expressed interest in utilizing it if better informed.^81^ Medication review national trends show a 3.5-fold increase between 2010 and 2015 with high physician acceptance rates, yet absolute implementation rates remain low.^82^ National collaborative pharmacy surveys in 2023 documented the economic benefits of pharmacist interventions including inquiry-based prescription modifications and home pharmaceutical care, but also highlighted the limited scale of these activities.^80^ (Table 3).

**Table 3:** Japan's implementation gap quantified.

Metric	Japan	International comparator (illustrative; not directly comparable)	Source	CFIR 2.0 domain
Prescribing–dispensing separation rate	80.3% (FY2023)	UK, Canada: ∼100% (historical); Korea: mandatory since 2000	^7^	OS (Policies & Laws)
Family pharmacist consent rate	2.90%	–	^80^	OS/IN (Local Attitudes; Motivation)
HSP public awareness	12.20%	–	^12^	OS (Local Attitudes)
Family pharmacist public awareness	22.2% (interest if informed: 40.7%)	–	^81^	OS (Local Attitudes)
Medication follow-up rate	0.84% of eligible patients	UK NMS: ∼1.1 M completed annually^a^	^10^	IP (Executing)
Pharmacist-led deprescribing rate	0.08%	–	^11^	IN (Motivation); IV (Complexity)
Mortality risk (family pharmacist)	Associated with ∼9% lower risk (CV/endocrine)	–	^76^	IV (Evidence Base)
TDDI prevention (family pharmacist, underage individuals)	Associated with lower incidence (IRR 1.3)	–	^77^	IV (Evidence Base)
Prescription modification rate (family vs. non-family pharmacist)	1.37× higher for family pharmacists	–	^78^	IN (Capability); IV (Relative Advantage)
Medication review trend (2010–2015)	3.5-fold increase; high physician acceptance	–	^82^	IP (Reflecting & Evaluating)
Alberta clinical service claims^b^	–	30,000 → 170,000/month (2012–2016)	^42^	OS (Financing)
UK Pharmacy First consultations^b^	–	Estimated capacity: up to 10 M GP appointments/year; ∼9900 participating pharmacies	^28^	OS (Policies & Laws)

Abbreviations: CV, cardiovascular; HSP, Health Support Pharmacy; IN, Individuals; IP, Implementation Process; IRR, incidence rate ratio; IV, Innovation; NMS, New Medicine Service; OS, Outer Setting; TDDI, therapeutic drug–drug interaction. a Based on NHSBSA dispensing data; direct percentage comparison with Japan's metric not available due to differences in denominator definition. b International comparator data included to illustrate scale of clinical service delivery in Tier 1 countries; direct percentage comparisons are not possible due to differences in metrics and denominators. Note: The international comparator column is illustrative only. Because of differences in denominators, metric definitions, and health-system structures, the Japanese and international figures are not directly numerically equivalent and should not be read as head-to-head comparisons. Dashes (—) indicate that directly comparable international data were not identified. Japan-specific metrics are derived from national survey and claims database studies.

### Cross-cutting educational dimension

The implementation barriers described above have an educational dimension that spans individual capability and system-level workforce development. A comparative study of community pharmacy education and practice between Japan and the United States identified structural differences in clinical training intensity, experiential learning opportunities, and patient interaction skills that contribute to the confidence and competency gaps observed among Japanese pharmacists.^83^

## Implementation strategies

The comparative analysis and Japan-specific findings described above point toward a set of evidence-informed implementation strategies, organized here using CFIR 2.0 domains. In presenting these strategies, we distinguish three bases for each recommendation: direct evidence from studies conducted within Japan; extrapolation from comparator countries whose institutional and cultural context differs from Japan's; and the authors' interpretive synthesis. Because no Japanese trials have directly evaluated most of the structural reforms proposed here, the majority of recommendations rest on extrapolation and interpretation rather than on direct domestic evidence, and should be read as hypotheses to guide policy experimentation rather than as established solutions.

Regarding Outer Setting interventions, the most consistent finding across Tier 1 countries is that payment reform is a necessary precondition for clinical service transformation. Alberta's compensation plan demonstrated that remunerating clinical services at rates comparable to physician consultations generated dramatic increases in service delivery.^42^ The United Kingdom's progressive shift from dispensing-based to service-based contractual frameworks provides a model for incremental payment reform within a centralized UHC system.^27^ For Japan, this points to the need for restructuring the fee schedule to create meaningful financial incentives for medication follow-up, deprescribing, and collaborative care services, while simultaneously reducing the relative attractiveness of high-volume dispensing (extrapolated from Tier 1 evidence27., 42; no direct Japanese evidence).

Inner Setting strategies require addressing the workforce infrastructure gap. The establishment of a formal pharmacy technician role, which exists in virtually all Tier 1 and many Tier 2 comparator countries but is absent in Japan,^70^ would enable task redistribution and free pharmacist time for clinical activities (extrapolated from cross-national workforce comparison^70^; the proposed benefit for Japan is an interpretive inference). Practice facilitation, demonstrated through a Canadian randomized controlled study to improve clinical service adoption,^48^ offers an evidence-based approach to supporting individual pharmacies through the transition process. Australia's experience with CFIR-informed implementation priority setting^38^ and ERIC-based facilitation strategies^37^ provides practical methodologies for identifying and addressing practice-level barriers.

At the Individuals level, strategies must address the capability, opportunity, and motivation dimensions identified through COM-B analysis. Clinical competency development should be aligned with international frameworks, as the FIP Global Competency Framework gap analysis suggests.^73^ Assertiveness training and interprofessional communication skills development may help address the cultural determinants of low clinical intervention rates (interpretive inference informed by the association between assertiveness and deprescribing experience reported in Japan^68^). The contrast between Japan's pharmacist confidence levels and those of international counterparts^74^ suggests that experiential training models—such as those integrated into Canadian pharmacy education—warrant adaptation for the Japanese context.

Innovation-level strategies should focus on reducing complexity and demonstrating relative advantage. Systematic reviews have shown that intervention complexity is a significant determinant of implementation success and that adaptation strategies are essential.^26^ Taiwan's PharmaCloud system illustrates how digital infrastructure can reduce the information asymmetry that makes clinical interventions more complex in fragmented information environments^54^, ^55^ (extrapolated from Taiwan's single-payer context, which differs substantially from Japan's). The economic evidence base for community pharmacy services, synthesized through cost-utility analyses, provides important justification for investment in transformation.^84^

Implementation Process strategies should emphasize systematic needs assessment and adaptive evaluation. The fragmented nature of Japan's reform efforts—multiple simultaneous policy changes without coordinated implementation support—contrasts with the more systematic approaches observed in Tier 1 countries. The vaccination service implementation literature, systematically analyzed through CFIR, demonstrates the value of structured barrier and facilitator assessment prior to service rollout^85^ (extrapolated from international implementation studies). (Fig. 4).

**Fig. 4:**
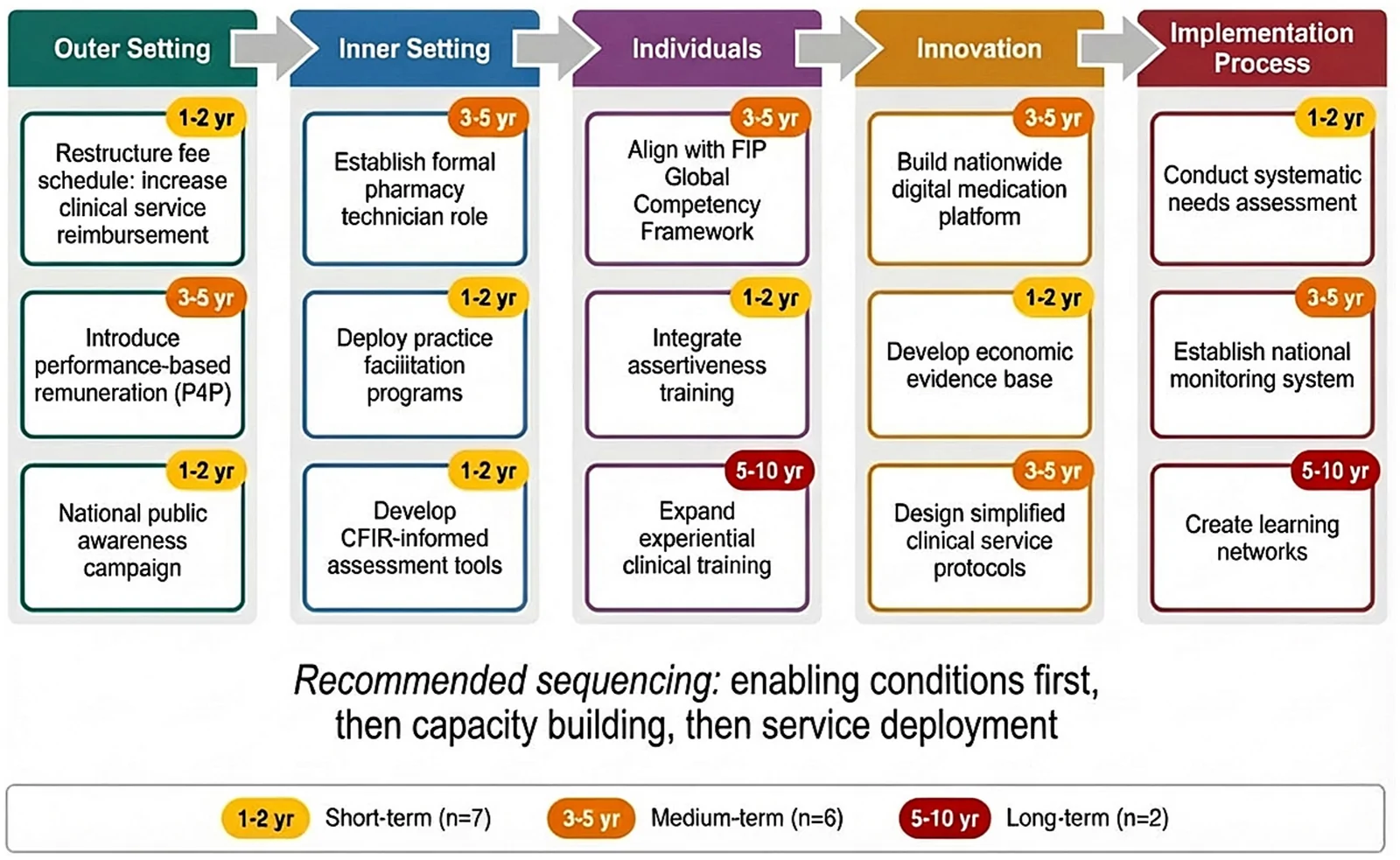
Evidence-informed implementation strategy roadmap for Japan's community pharmacy transformation, organized by CFIR 2.0 domains. This roadmap represents a proposed, evidence-informed framework rather than an empirically validated or proven implementation strategy. The sequencing and timeframes are analytical suggestions derived from cross-national comparison and are intended to guide policy experimentation rather than to prescribe a definitive implementation pathway. Five columns represent the sequential CFIR 2.0 domains from enabling conditions (left) to service deployment and evaluation (right): Outer Setting, Inner Setting, Individuals, Innovation, and Implementation Process. Each column contains two to three recommended actions derived from the cross-national comparative analysis. Actions are classified by implementation timeframe: short-term (1–2 years; yellow), medium-term (3–5 years; orange), and long-term (5–10 years; red). The roadmap emphasizes that system-level enabling conditions (payment reform, workforce legislation) should precede individual-level capacity building, which in turn should precede large-scale service innovation and process evaluation. CFIR 2.0, updated Consolidated Framework for Implementation Research; FIP, International Pharmaceutical Federation; HSP, Health Support Pharmacy; P4P, pay-for-performance. International evidence sources supporting each recommended action: Outer Setting—restructure fee schedule,^42^, ^47^ performance-based remuneration,27., 32 public awareness campaign^12^, 81.; Inner Setting—pharmacy technician role,^70^ practice facilitation,^48^ CFIR-informed assessment^37^, ^38^; Individuals—FIP competency alignment,^73^ assertiveness training,^68^ experiential clinical training^74^, ^83^; Innovation—digital medication platform,^54^, ^55^ economic evidence base,^76^, ^84^ simplified service protocols^26^; Implementation Process—systematic needs assessment,^85^ national monitoring system,^20^, ^64^ learning networks.^48^ This image was created with assistance of Claude (Anthropic). (For interpretation of the references to colour in this figure legend, the reader is referred to the web version of this article.)

## Discussion

This review reveals that Japan's community pharmacy transformation challenge is neither purely institutional nor purely cultural but rather emerges from the interaction between the two. The CFIR 2.0 framework proved particularly useful for capturing this interaction because its updated constructs—especially Local Attitudes, Recipient-Centeredness, and COM-B-based Characteristics—explicitly accommodate cultural factors that are often treated as external to implementation analysis.

The comparison across three tiers of countries shows several cross-cutting themes. First, structural reform without cultural and behavioral adaptation is insufficient. Japan's achievement of dispensing separation, like Korea's,^49^ demonstrates that structural change does not automatically translate into functional transformation. Second, payment reform is necessary but not sufficient. While Alberta's experience^42^ and the UK's contractual evolution^27^ confirm the importance of aligned financial incentives, an analysis of Australia's CPA suggests that remuneration agreements designed to support new roles may not effectively advance professional scope if their financial distribution and structural design continue to prioritize traditional dispensing models over clinical service development.^36^ Third, scope-of-practice expansion requires parallel investment in interprofessional relationships. The persistent tensions between pharmacists and physicians documented in the UK,^33^ Singapore,^60^ and Korea^52^ suggest that legislative authority must be accompanied by relational infrastructure.

The COVID-19 pandemic served as a natural experiment in pharmacy scope expansion, with multiple countries granting temporary or permanent extensions to pharmacist authority.^86^ Australia's policy analysis of COVID-related scope changes^87^ and Taiwan's demonstration of pharmacy network public health utility^58^ illustrate how critical incidents can catalyze reforms that were previously politically unfeasible. For Japan, the pandemic period did not result in comparable scope expansion. Japanese pharmacists were not included in the temporary legal authorizations to administer COVID-19 vaccines,^21^ in contrast to the expanded pandemic roles granted to community pharmacists in several UHC systems, most extensively documented in the United Kingdom.^4^, 86. This absence of comparable scope expansion in Japan reflects the institutional and regulatory constraints identified across CFIR 2.0 domains. Beyond these specific policy catalysts, deeper cultural and institutional factors shape the pace and direction of pharmacy transformation.

Cultural dimensions have received relatively little attention in implementation research and warrant further examination. Japan's cultural profile—characterized by high uncertainty avoidance, moderate power distance, and mid-range collectivism—may be one of several factors that, alongside reimbursement structures, legal authority, and workforce configuration, contribute to the persistence of existing practice patterns.^15^ The assertiveness findings,^68^ the deprescribing reluctance patterns,^69^, 75. and the low proactive intervention rates^10^, ^11^ are all consistent with this cultural interpretation. Culture, however, should not be treated as a fixed or deterministic barrier: Singapore's physician-led, pharmacist-enabled model^59^ and Korea's Family Pharmacy concept^50^ represent culturally adapted approaches that work within rather than against hierarchical norms. Cultural dimensions operate alongside—and are shaped by—policy, reimbursement, legal authority, workforce structure, and professional norms; attributing implementation gaps primarily to national culture would overstate the evidence and risk essentializing a dynamic and changeable set of professional behaviors.

This review has several limitations. First, as a narrative review, the literature selection involved subjective judgment, and the analysis does not claim to be exhaustive; moreover, screening was conducted by a single reviewer (RU), which may introduce selection bias. Second, the non-inclusion of Embase and limited grey literature searching may have resulted in relevant publications being missed. Third, the breadth of the comparative scope necessarily limits the depth of analysis for individual countries. Fourth, many Japanese-language sources in Ichushi-Web lacked English abstracts, requiring reliance on title-based assessment for initial screening. Fifth, the evidence-driven depth approach means that countries with more extensive English-language research literature (particularly the United Kingdom and Canada) receive more detailed treatment than those with less accessible evidence bases. Sixth, CFIR 2.0 was applied as a post hoc analytical framework to organize findings from studies that were not originally designed using this framework; consequently, the mapping of findings to specific CFIR 2.0 constructs involved interpretive judgment by the authors.

## Conclusions

Japan's experience with community pharmacy transformation under universal health coverage provides important lessons for health systems worldwide. The achievement of structural prescribing–dispensing separation, while necessary, is insufficient to drive the transition from product-centered to patient-centered pharmacy practice. We identified implementation barriers across all five CFIR 2.0 domains, with cultural factors appearing to interact with institutional structures in ways that slow the pace of transformation and limit the effectiveness of incremental, non-coordinated reform efforts.

The emerging outcome evidence for Japan's family pharmacist system suggests promising effectiveness when delivered, making the implementation gap—rather than the innovation design—the primary target for intervention. Cross-national comparison reveals that successful transformation requires simultaneous action on payment reform, scope-of-practice legislation, workforce restructuring, interprofessional relationship building, and culturally sensitive implementation support. The updated CFIR 2.0 framework, with its explicit attention to local attitudes, recipient-centeredness, and individual characteristics, provides a robust analytical tool for understanding and addressing the culturally embedded nature of pharmacy transformation barriers in UHC systems.

## Declaration of generative AI and AI-assisted technologies in the manuscript preparation process

During the preparation of this work the authors used Claude (Anthropic) in order to assist with drafting, language editing, structural formatting of the manuscript, and preparation of Fig. 3, Fig. 4 under the authors' direction. After using this tool, the authors reviewed and edited the content as needed and take full responsibility for the content of the published article.

## CRediT authorship contribution statement

**Ryuta Urakawa:** Writing – review & editing, Writing – original draft, Visualization, Validation, Supervision, Project administration, Methodology, Investigation, Data curation, Conceptualization. **Kazumi Kubota:** Writing – review & editing, Validation, Conceptualization. **Risa Marikawa Urakawa:** Writing – review & editing, Conceptualization. **Rika Tanaka:** Writing – review & editing, Conceptualization.

## Funding

There was no funding granted for this article.

## Declaration of competing interest

The authors declare that they have no competing interests.

## Data Availability

All data generated or analyzed during this study are included in this published article and its supplementary file. Search strategies are detailed in the Methods section and Supplementary File 1.

## Appendix A Supplementary data

Supplementary material Supplementary File 1 Detailed search strategies, database results, and screening process for the narrative review.

## References

[1] World Health Organization (2007).

[2] World Health Organization, World Bank (2025).

[3] Rose O., Derendorf H., Erzkamp S. (2018). Development of clinical pharmacy services in Australia, Austria, Belgium, Bosnia-Herzegovina, Canada, Germany, Japan, Kosovo, Switzerland, the Netherlands, Thailand, USA and correlation with educational standards, level of research, and implementation practices. Int J Clin Pharmacol Ther.

[4] Jordan D., Guiu-Segura J.M., Sousa-Pinto G., Wang L.N. (2021). How COVID-19 has impacted the role of pharmacists around the world. Farm Hosp.

[5] Taketate K., Akagi K. (2024). Nihon no yakuzai-shi no tachii-chi to yakuwari no hensen: Meiji kara genzai made no 150-nenshi [Changes in the position and role of Japanese pharmacists: 150 years of history from Meiji to the present]. Jpn J Hist Pharm.

[6] Shishido M. (2019). Yakkyoku yakuzaishi ni motomerareru yakuwari no hensen to genzai no giron [The changing role of the community pharmacist and current discussions]. Refarensu.

[7] Ministry of Health, Labour and Welfare (2025). https://www.mhlw.go.jp/wp/hakusyo/kousei/25-2/dl/02.pdf.

[8] Ministry of Health, Labour and Welfare (2015). https://www.mhlw.go.jp/stf/houdou/0000102179.html.

[9] Takamura K., Tachibana K., Kihira T., Nakai K., Tsutsumi Y., Kondoh M. (2021). Legislation on the roles of the pharmacist and pharmacy in the revision of the pharmaceutical and medical device act and the pharmacists act in Japan. Ther Innov Regul Sci.

[10] Sekiya S., Tanaka R., Iijima H. (2024). A nationwide survey on medication follow-up care by community pharmacists: from the Japanese nationwide pharmacy collaboration survey in 2023. Yakugaku Zasshi.

[11] Fujita K., Sato N., Okizaki A. (2026). Pharmacist-led deprescribing interventions for older adults with polypharmacy: a retrospective cohort study of community pharmacy practice in Japan. Drugs Aging.

[12] Hirota N., Okamura N. (2020). Patients’ attitudes, awareness, and opinions about community pharmacies in Japan: next steps for the health support pharmacy system. Integr Pharm Res Pract.

[13] Costa F.A., Scullin C., Al-Taani G. (2017). Provision of pharmaceutical care by community pharmacists across Europe: is it developing and spreading?. J Eval Clin Pract.

[14] Hofstede G., Hofstede G.J., Minkov M. (2010).

[15] Braithwaite J., Tran Y., Ellis L.A., Westbrook J. (2020). Inside the black box of comparative national healthcare performance in 35 OECD countries: issues of culture, systems performance and sustainability. PloS One.

[16] Damschroder L.J., Reardon C.M., Widerquist M.A.O., Lowery J. (2022). The updated consolidated framework for implementation research based on user feedback. Implement Sci.

[17] Ferrari R. (2015). Writing narrative style literature reviews. Med Writ.

[18] Greenhalgh T., Thorne S., Malterud K. (2018). Time to challenge the spurious hierarchy of systematic over narrative reviews?. Eur J Clin Invest.

[19] Grant M.J., Booth A. (2009). A typology of reviews: an analysis of 14 review types and associated methodologies. Health Info Libr J.

[20] Damschroder L.J., Reardon C.M., Opra Widerquist M.A., Lowery J. (2022). Conceptualizing outcomes for use with the consolidated framework for implementation research (CFIR): the CFIR outcomes addendum. Implement Sci.

[21] Yongue J.S. (2024). Reflections on the role of the Japanese pharmacist during the COVID-19 pandemic: lessons from the history of pharmacy. Jpn J Hist Pharm.

[22] Page M.J., McKenzie J.E., Bossuyt P.M. (2021). The PRISMA 2020 statement: an updated guideline for reporting systematic reviews. BMJ.

[23] Shoemaker S.J., Curran G.M., Swan H., Teeter B.S., Thomas J. (2017). Application of the consolidated framework for implementation research to community pharmacy: a framework for implementation research on pharmacy services. Res Social Adm Pharm.

[24] Weir N.M., Newham R., Dunlop E., Bennie M. (2019). Factors influencing national implementation of innovations within community pharmacy: a systematic review applying the consolidated framework for implementation research. Implement Sci.

[25] Pereira C.E.O., Bambirra E.H.F., Fernandes B.D., Sousa M.C.V.B., Mendonça S.A.M., Chemello C. (2022). Factors influencing the implementation of pharmaceutical care in outpatient settings: a systematic review applying the consolidated framework for implementation research. Res Social Adm Pharm.

[26] Moecker R., Terstegen T., Haefeli W.E., Seidling H.M. (2021). The influence of intervention complexity on barriers and facilitators in the implementation of professional pharmacy services—a systematic review. Res Social Adm Pharm.

[27] Anderson C., Sharma R. (2020). Primary health care policy and vision for community pharmacy and pharmacists in England. Pharm Pract (Granada).

[28] Glover R.E., Lalani M., Sonnex K. (2025). A mixed methods protocol for an impact and implementation evaluation of the pharmacy first services for management of common conditions in England. Int J Pharm Pract.

[29] Edwards J., Coward M., Carey N. (2022). Barriers and facilitators to implementation of non-medical independent prescribing in primary care in the UK: a qualitative systematic review. BMJ Open.

[30] Elliott R.A., Boyd M.J., Salema N.E. (2016). Supporting adherence for people starting a new medication for a long-term condition through community pharmacies: a pragmatic randomised controlled trial of the new medicine service. BMJ Qual Saf.

[31] Latif A., Waring J., Watmough D. (2016). Examination of England’s new medicine service (NMS) of complex health care interventions in community pharmacy. Res Social Adm Pharm.

[32] Schafheutle E.I., Moss A.A., Hindi A.M.K. (2025). Do pay-for-performance schemes improve quality in community pharmacy? A mixed-methods study exploring stakeholder perspectives on implementation of the nationwide pharmacy quality scheme (PQS) in England. PloS One.

[33] Hindi A.M.K., Jacobs S., Schafheutle E.I. (2019). Solidarity or dissonance? A systematic review of pharmacist and GP views on community pharmacy services in the UK. Health Soc Care Community.

[34] Jacobs S., Bradley F., Elvey R. (2017).

[35] Dineen-Griffin S., Benrimoj S.I., Garcia-Cardenas V. (2020). Primary health care policy and vision for community pharmacy and pharmacists in Australia. Pharm Pract (Granada).

[36] Jackson J.K., Scahill S.L., Mintrom M., Kirkpatrick C.M. (2023). An evaluation of the Australian community pharmacy agreement from a public policy perspective: industry policy cloaked as health policy?. J Pharm Policy Pract.

[37] Moullin J.C., Sabater-Hernández D., Benrimoj S.I. (2016). Qualitative study on the implementation of professional pharmacy services in Australian community pharmacies using framework analysis. BMC Health Serv Res.

[38] Seda V., Carter S.R., Moles R.J., Schneider C.R. (2025). Implementation priorities in Australian community pharmacy: a semi-structured survey of Australian pharmacists. Explor Res Clin Soc Pharm.

[39] Jokanovic N., Tan E.C., van den Bosch D., Kirkpatrick C.M., Dooley M.J., Bell J.S. (2016). Clinical medication review in Australia: a systematic review. Res Social Adm Pharm.

[40] Yong F.R., Hor S.Y., Bajorek B.V. (2021). Considerations of Australian community pharmacists in the provision and implementation of cognitive pharmacy services: a qualitative study. BMC Health Serv Res.

[41] Raiche T., Pammett R., Dattani S. (2020). Community pharmacists' evolving role in Canadian primary health care: a vision of harmonization in a patchwork system. Pharm Pract (Granada).

[42] Breault R.R., Whissell J.G., Hughes C.A., Schindel T.J. (2017). Development and implementation of the compensation plan for pharmacy services in Alberta, Canada. J Am Pharm Assoc.

[43] Faruquee C.F., Khera A.S., Guirguis L.M. (2020). Family physicians’ perceptions of pharmacists prescribing in Alberta. J Interprof Care.

[44] Tsuyuki R.T., Al Hamarneh Y.N., Jones C.A., Hemmelgarn B.R. (2016). The effectiveness of pharmacist interventions on cardiovascular risk: the multicenter randomized controlled RxEACH trial. J Am Coll Cardiol.

[45] Clemens S., Eisl-Raudaschl L., Pachmayr J., Rose O. (2025). Community pharmacist prescribing: roles and competencies-a systematic review and implications. Pharmacy (Basel).

[46] Mesbahi Z., Piquer-Martinez C., Benrimoj S.I. (2025). Pharmacists as independent prescribers in community pharmacy: a scoping review. Res Social Adm Pharm.

[47] Ceka A., Juneja B., Tseng O. (2026). Bridging the gap between expanded pharmacy services and payment models: a jurisdictional scan. J Am Pharm Assoc.

[48] Houle S.K., Charrois T.L., Faruquee C.F., Tsuyuki R.T., Rosenthal M.M. (2017). A randomized controlled study of practice facilitation to improve the provision of medication management services in Alberta community pharmacies. Res Social Adm Pharm.

[49] Yuk S.M., Han K.T., Kim S.J. (2015). Consumption of pharmaceutical drugs in exception region of separation for drug prescribing and dispensing program in South Korea. Subst Abuse Treat Prev Policy.

[50] Yang D.W., Son K.B. (2025). Assessing public acceptance and perceived need for a “family pharmacy” model in South Korea: a cross-sectional web-based survey. Fam Pract.

[51] Son K.B., Choi S., Kim D. (2019). Public perceptions of the roles and functions of community pharmacies in the era of expanding scopes of pharmaceutical practice: a questionnaire survey in South Korea. Health Soc Care Community.

[52] Kim D.S., Bae G., Yoo S.Y., Kang M. (2015). Between government policy, clinical autonomy, and market demands: a qualitative study of the impact of the prescribing analysis system on behavior of physicians in South Korea. BMC Health Serv Res.

[53] Kim D.W., Lee B.J. (2022). Recognition investigation of community pharmacists implementing good pharmacy practice in Korea. Int J Health Serv.

[54] Huang S.K., Wang P.J., Tseng W.F. (2015). NHI-PharmaCloud in Taiwan–a preliminary evaluation using the RE-AIM framework and lessons learned. Int J Med Inform.

[55] Lin J.H., Cheng S.H. (2020). The impact of a national health information exchange program under a single-payer system. Med Care.

[56] Hsu J.C., Ross-Degnan D., Wagner A.K. (2015). Utilization of oral antidiabetic medications in Taiwan following strategies to promote access to medicines for chronic diseases in community pharmacies. J Pharm Policy Pract.

[57] Huang Y.Y., Cheng S.H. (2019). A community pharmacist home visit project for high utilizers under a universal health system: a preliminary assessment. Health Policy.

[58] Lin C.H., Lin Y.W., Wang J.Y., Lin M.H. (2020). The pharmaceutical practice of mask distribution by pharmacists in Taiwan’s community pharmacies under the mask real-name system, in response to the COVID-19 outbreak. Cost Eff Resour Alloc.

[59] Fletcher D.B., Loh G.L., Chang Shu-Wen G. (2025). Advancing cardiology pharmacy practice: a position paper on physician-led, pharmacist-enabled model for sustainable cardiovascular care. Ann Acad Med Singapore.

[60] Chong J.B.K., Yap C.Y.H., Tan S.L.L., Thong X.R., Fang Y., Smith H.E. (2023). General practitioners’ perceptions of the roles of community pharmacists and their willingness to collaborate with pharmacists in primary care. J Pharm Policy Pract.

[61] Low H.M.M., See Z.Y., Lai Y.F. (2020). Understanding and expectation towards pharmaceutical care among patients, caregivers and pharmacy service providers: a qualitative study. Eur J Hosp Pharm.

[62] Hohmeier K.C., Renfro C., Turner K. (2021). The Tennessee Medicaid medication therapy management program: early stage contextual factors and implementation outcomes. BMC Health Serv Res.

[63] Newman T.V., Hernandez I., Keyser D. (2019). Optimizing the role of community pharmacists in managing the health of populations: barriers, facilitators, and policy recommendations. J Manag Care Spec Pharm.

[64] Amayreh R., Shah S., Goode J.K. (2026). Implementation of statewide protocols for community-based pharmacist services in Virginia: a qualitative study using the consolidated framework for implementation research. J Am Pharm Assoc.

[65] Westerlund T., Marklund B. (2020). Community pharmacy and primary health care in Sweden - at a crossroads. Pharm Pract (Granada).

[66] Airaksinen M., Toivo T., Jokinen L. (2021). Policy and vision for community pharmacies in Finland: a roadmap towards enhanced integration and reduced costs. Pharm Pract (Granada).

[67] Takada M. (2016). Shin no kokumin no tame no iyaku bungyo towa: Nihon no iyaku bungyo seisaku, chozai hoshu no mujun to kadai [What is the genuine separation of dispensing and prescribing for citizens? Contradictions and problems of Japan's separation policy and dispensing fees]. Inochi to Kurashi Kenkyusho Ho.

[68] Ishii M., Ozone S., Masumoto S., Maeno T. (2025). Assertiveness in community pharmacists and their experience of pharmacist-led deprescribing: a cross-sectional study. Res Social Adm Pharm.

[69] Ie K., Machino R., Albert S.M. (2023). Deprescribing as an opportunity to facilitate patient-centered care: a qualitative study of general practitioners and pharmacists in Japan. Int J Environ Res Public Health.

[70] Koehler T., Brown A. (2017). A global picture of pharmacy technician and other pharmacy support workforce cadres. Res Social Adm Pharm.

[71] Katsuta D., Ohashi Y., Kamimura M. (2024). Status of community pharmacy functions and regional medical collaboration system initiatives in Japan. Biol Pharm Bull.

[72] Sato N., Fujita K., Kushida K., Chen T.F. (2020). Exploring the factors influencing the quality of “health support pharmacy” services in Japan: perspectives of community pharmacists. Res Social Adm Pharm.

[73] Arakawa N., Yamamura S., Duggan C., Bates I. (2020). The development of a foundation-level pharmacy competency framework: an analysis of country-level applicability of the global competency framework. Res Social Adm Pharm.

[74] Mittal S., Okada H., Bermingham M. (2023). Community pharmacists’ attitude, practice and confidence in supporting people with diabetes in Japan and Ireland: a cross-sectional survey. Yakugaku Zasshi.

[75] Kumaki R., Goto H., Akagawa K., Goto T., Kishimoto K. (2023). Yakkyoku yakuzaishi ga kangaeru genyaku teian shitakutemo dekinai riyu to sono kaizensaku no chosa [Survey on reasons why community pharmacists cannot propose deprescribing despite wanting to, and improvement measures]. Nihon Ronen Yakugaku Kaishi.

[76] Iketani R., Imai S. (2026). Family pharmacist system for patients with chronic cardiovascular or endocrine disease. JAMA Netw Open.

[77] Iketani R. (2025). Effectiveness of the kakaritsuke-yakuzaishi (family pharmacist) system for underage individuals in Japan: a cohort study using a health insurance claims database. BMC Health Serv Res.

[78] Nishikawa T., Sakata N., Sugiyama T., Tamiya N. (2023). Dispensing by family pharmacists as a potential factor in pharmacist-initiated prescription change: a retrospective observational study. J Epidemiol.

[79] Goto K., Sato T., Shikamura Y., Nomura K., Negishi K., Hanawa T. (2020). Examining the association between the “my pharmacist” model and the service quality of community pharmacies. Res Social Adm Pharm.

[80] Sekiya S., Tanaka R., Iijima H. (2025). Prevention of serious adverse drug reactions and economic effects through community pharmacists’ inquiries, home care, and medication follow-up: from the Japanese nationwide pharmacy collaboration survey in 2023. Yakugaku Zasshi.

[81] Ishimura J., Tomisaki M. (2022). "Kakaritsuke yakuzaishi" seido ni kansuru kanja ishiki chosa [Patient awareness survey on the "family pharmacist" system]. Yakkyoku Yakugaku.

[82] Koyama T., Onoue H., Ohshima A. (2018). Trends in the medication reviews of community pharmacies in Japan: a nationwide retrospective study. Int J Clin Pharm.

[83] Hasumoto K.Y., Thomas R.K., Yokoi M., Arai K. (2020). Comparison of community pharmacy practice in Japan and US state of Illinois. J Pharm Pract.

[84] Sanyal C., Husereau D.R. (2019). Community-based services by pharmacists: a systematic review of cost-utility analyses. Value Health.

[85] Abdulselam M., El-Awaisi A., Nasr Z.G., Shaar S., El Hajj M.S. (2025). Barriers and facilitators for pharmacist-led vaccination services: a systematic review using the consolidated framework for implementation research (CFIR). Explor Res Clin Soc Pharm.

[86] Merks P., Jakubowska M., Drelich E. (2021). The legal extension of the role of pharmacists in light of the COVID-19 global pandemic. Res Social Adm Pharm.

[87] Chiu K., Thow A.M., Bero L. (2022). “Never waste a good crisis”: opportunities and constraints from the COVID-19 pandemic on pharmacists’ scope of practice. Res Social Adm Pharm.

