# Supplementary material for: Community pharmacy transformation under universal health coverage: Implementation barriers, cultural determinants, and cross-national lessons from Japan's ongoing transition – A narrative review
Source: Explor Res Clin Soc Pharm. 2026 Sep 20;24:100850. doi: 10.1016/j.rcsop.2026.100850 (PMC13631658; doi:10.1016/j.rcsop.2026.100850)
Supplement: Supplementary file 1 [file mmc1.docx]

**Title:** Detailed search strategies, database results, and screening process for the narrative review

**1. Overview**

This additional file provides the complete search strategies used for each database and search category, the number of records retrieved, the deduplication process, and the screening criteria applied. Searches were conducted on February 26, 2026.

**2. Database search strategies**

**Search 1: Japan's community pharmacy context**

**PubMed/MEDLINE**: 130 records

("Community Pharmacy Services"[MeSH Terms] OR "Pharmacies"[MeSH Terms] OR

"Pharmacists"[MeSH Terms] OR "community pharmac*"[Title/Abstract] OR

"community pharmacist*"[Title/Abstract] OR "retail pharmac*"[Title/Abstract] OR

"dispensing pharmac*"[Title/Abstract])

AND

("Japan"[MeSH Terms] OR "Japan"[Title/Abstract] OR "Japanese"[Title/Abstract])

AND

("Pharmaceutical Services"[MeSH Terms] OR "Medication Therapy Management"[MeSH Terms] OR

"Primary Health Care"[MeSH Terms] OR "Home Care Services"[MeSH Terms] OR

"Polypharmacy"[MeSH Terms] OR "pharmaceutical care"[Title/Abstract] OR

"medication management"[Title/Abstract] OR "medication review"[Title/Abstract] OR

"pharmacist role*"[Title/Abstract] OR "scope of practice"[Title/Abstract] OR

"pharmacy practice"[Title/Abstract] OR

"separation of prescribing and dispensing"[Title/Abstract] OR

"iyaku-bungyo"[Title/Abstract] OR "kakaritsuke"[Title/Abstract] OR

"polypharmacy"[Title/Abstract] OR "home visit*"[Title/Abstract] OR

"health support pharmacy"[Title/Abstract])

AND ("2016/01/01"[Date - Publication] : "2026/02/26"[Date - Publication])

AND English[Language]

**Ichushi-Web (Japan Medical Abstracts Society)**: 514 records

(薬局/TH OR 保険薬局/AL OR 調剤薬局/AL OR コミュニティファーマシー/AL OR

薬局薬剤師/AL)

AND

(かかりつけ薬剤師/AL OR 健康サポート薬局/AL OR 地域連携薬局/AL OR 在宅医療/TH OR

ポリファーマシー/AL OR 対人業務/AL OR 地域包括ケアシステム/TH OR 医薬分業/AL OR

セルフメディケーション/AL OR 薬剤師の役割/AL OR 多職種連携/AL)

AND (PT=原著論文 OR PT=解説 OR PT=総説)

AND (DT=2016:2026)

AND (CK=ヒト)

**Search 2: Tier 1 — Successful transformation models (United Kingdom, Australia, Canada)**

**PubMed/MEDLINE — United Kingdom**: 454 records

("Community Pharmacy Services"[MeSH Terms] OR "Pharmacies"[MeSH Terms] OR

"community pharmac*"[Title/Abstract] OR "community pharmacist*"[Title/Abstract])

AND

("United Kingdom"[MeSH Terms] OR "United Kingdom"[Title/Abstract] OR

"NHS"[Title/Abstract] OR "National Health Service"[Title/Abstract] OR

"England"[Title/Abstract] OR "Scotland"[Title/Abstract] OR "Wales"[Title/Abstract])

AND

("Pharmaceutical Services"[MeSH Terms] OR "clinical service*"[Title/Abstract] OR

"medication review*"[Title/Abstract] OR "minor ailment*"[Title/Abstract] OR

"medicines use review*"[Title/Abstract] OR "new medicine service*"[Title/Abstract] OR

"pharmacy first"[Title/Abstract] OR "extended role*"[Title/Abstract] OR

"pharmacist prescri*"[Title/Abstract] OR "public health role*"[Title/Abstract] OR

"scope of practice"[Title/Abstract] OR "pharmacist-led"[Title/Abstract] OR

"pharmacy service*"[Title/Abstract])

AND ("2016/01/01"[Date - Publication] : "2026/02/26"[Date - Publication])

AND English[Language]

**PubMed/MEDLINE — Australia**: 369 records

("Community Pharmacy Services"[MeSH Terms] OR "Pharmacies"[MeSH Terms] OR

"community pharmac*"[Title/Abstract] OR "community pharmacist*"[Title/Abstract])

AND

("Australia"[MeSH Terms] OR "Australia"[Title/Abstract] OR

"Australian"[Title/Abstract])

AND

("Pharmaceutical Services"[MeSH Terms] OR "medication review*"[Title/Abstract] OR

"MedsCheck"[Title/Abstract] OR "home medicines review*"[Title/Abstract] OR

"clinical intervention*"[Title/Abstract] OR

"community pharmacy agreement*"[Title/Abstract] OR

"pharmacist service*"[Title/Abstract] OR "extended role*"[Title/Abstract] OR

"scope of practice"[Title/Abstract] OR "pharmacist-led"[Title/Abstract] OR

"pharmacy service*"[Title/Abstract] OR "pharmacy practice"[Title/Abstract])

AND ("2016/01/01"[Date - Publication] : "2026/02/26"[Date - Publication])

AND English[Language]

**PubMed/MEDLINE — Canada**: 246 records

("Community Pharmacy Services"[MeSH Terms] OR "Pharmacies"[MeSH Terms] OR

"community pharmac*"[Title/Abstract] OR "community pharmacist*"[Title/Abstract])

AND

("Canada"[MeSH Terms] OR "Canada"[Title/Abstract] OR "Canadian"[Title/Abstract])

AND

("Pharmaceutical Services"[MeSH Terms] OR "clinical service*"[Title/Abstract] OR

"medication review*"[Title/Abstract] OR "minor ailment*"[Title/Abstract] OR

"pharmacist prescri*"[Title/Abstract] OR "scope of practice"[Title/Abstract] OR

"extended role*"[Title/Abstract] OR "pharmacy practice"[Title/Abstract] OR

"collaborative practice"[Title/Abstract] OR "pharmacist-led"[Title/Abstract] OR

"pharmacy service*"[Title/Abstract])

AND ("2016/01/01"[Date - Publication] : "2026/02/26"[Date - Publication])

AND English[Language]

**Search 3: Tier 2 — Asian comparators (Republic of Korea, Taiwan, Singapore)**

**PubMed/MEDLINE**: 300 records

("Community Pharmacy Services"[MeSH Terms] OR "Pharmacies"[MeSH Terms] OR

"Pharmacists"[MeSH Terms] OR "Pharmaceutical Services"[MeSH Terms] OR

"community pharmac*"[Title/Abstract] OR "community pharmacist*"[Title/Abstract] OR

"pharmacy practice"[Title/Abstract] OR "pharmaceutical care"[Title/Abstract] OR

"pharmacist role*"[Title/Abstract] OR "pharmacist* service*"[Title/Abstract] OR

"retail pharmac*"[Title/Abstract])

AND

("Republic of Korea"[MeSH Terms] OR "Korea"[Title/Abstract] OR

"Korean"[Title/Abstract] OR "Taiwan"[MeSH Terms] OR "Taiwan"[Title/Abstract] OR

"Taiwanese"[Title/Abstract] OR "Singapore"[MeSH Terms] OR

"Singapore"[Title/Abstract])

AND

("Health Policy"[MeSH Terms] OR "National Health Programs"[MeSH Terms] OR

"Medication Therapy Management"[MeSH Terms] OR

"Primary Health Care"[MeSH Terms] OR

"separation of prescribing and dispensing"[Title/Abstract] OR

"medication management"[Title/Abstract] OR "health system*"[Title/Abstract] OR

"health polic*"[Title/Abstract] OR "pharmacy reform*"[Title/Abstract] OR

"clinical service*"[Title/Abstract] OR "extended role*"[Title/Abstract] OR

"scope of practice"[Title/Abstract] OR "implementation"[Title/Abstract] OR

"national health insurance"[Title/Abstract] OR

"universal health coverage"[Title/Abstract])

AND ("2011/01/01"[Date - Publication] : "2026/02/26"[Date - Publication])

AND English[Language]

**Search 4: CFIR framework and implementation science**

**PubMed/MEDLINE**: 224 records

("Consolidated Framework for Implementation Research"[Title/Abstract] OR

"CFIR"[Title/Abstract])

AND

("Pharmacies"[MeSH Terms] OR "Community Pharmacy Services"[MeSH Terms] OR

"Pharmacists"[MeSH Terms] OR "Pharmaceutical Services"[MeSH Terms] OR

"community pharmac*"[Title/Abstract] OR "community pharmacist*"[Title/Abstract] OR

"pharmacist*"[Title/Abstract] OR "pharmaceutical care"[Title/Abstract] OR

"pharmacy service*"[Title/Abstract] OR "pharmacy practice"[Title/Abstract])

AND ("2011/01/01"[Date - Publication] : "2026/02/26"[Date - Publication])

AND English[Language]

**Search 5: Tier 3 — United States MTM**

**PubMed/MEDLINE**: 117 records

("Medication Therapy Management"[MeSH Terms] OR

"medication therapy management"[Title/Abstract] OR "MTM"[Title/Abstract] OR

"comprehensive medication management"[Title/Abstract] OR "CMM"[Title/Abstract])

AND

("United States"[MeSH Terms])

AND

("Pharmacies"[MeSH Terms] OR "Community Pharmacy Services"[MeSH Terms] OR

"community pharmac*"[Title/Abstract] OR "ambulatory"[Title/Abstract] OR

"outpatient"[Title/Abstract])

AND

(outcome*[Title/Abstract] OR effectiveness[Title/Abstract] OR

implementation[Title/Abstract] OR barrier*[Title/Abstract] OR

facilitator*[Title/Abstract] OR evaluation[Title/Abstract])

AND ("2016/01/01"[Date - Publication] : "2026/02/26"[Date - Publication])

AND English[Language]

**Search 6: Tier 3 — Nordic countries**

**PubMed/MEDLINE**: 142 records

("community pharmac*"[Title/Abstract] OR "community pharmacist*"[Title/Abstract] OR

"Community Pharmacy Services"[MeSH Terms] OR "Pharmacies"[MeSH Terms] OR

"Pharmacists"[MeSH Terms])

AND

("Sweden"[MeSH Terms] OR "Denmark"[MeSH Terms] OR "Norway"[MeSH Terms] OR

"Finland"[MeSH Terms] OR "Nordic"[Title/Abstract] OR

"Scandinavian"[Title/Abstract] OR "Sweden"[Title/Abstract] OR

"Denmark"[Title/Abstract] OR "Norway"[Title/Abstract] OR

"Finland"[Title/Abstract])

AND

("integrated care"[Title/Abstract] OR "self-care"[Title/Abstract] OR

"self care"[Title/Abstract] OR "prevention"[Title/Abstract] OR

"pharmacist role*"[Title/Abstract] OR "extended role*"[Title/Abstract] OR

"collaboration"[Title/Abstract] OR "e-health"[Title/Abstract] OR

"ehealth"[Title/Abstract] OR "e-prescription*"[Title/Abstract] OR

"electronic prescri*"[Title/Abstract] OR "pharmacy service*"[Title/Abstract] OR

"clinical service*"[Title/Abstract] OR "medication review*"[Title/Abstract] OR

"scope of practice"[Title/Abstract])

AND ("2011/01/01"[Date - Publication] : "2026/02/26"[Date - Publication])

AND English[Language]

**3. Record retrieval and deduplication**

Table S1 summarizes the records retrieved from each search and the deduplication results.

**Table S1.** Summary of database search results and deduplication

| **Search** | **Database** | **Records retrieved** |
| --- | --- | --- |
| 1: Japan | PubMed/MEDLINE | 130 |
| 1: Japan | Ichushi-Web | 514 |
| 2: Tier 1 — UK | PubMed/MEDLINE | 454 |
| 2: Tier 1 — Australia | PubMed/MEDLINE | 369 |
| 2: Tier 1 — Canada | PubMed/MEDLINE | 246 |
| 3: Tier 2 — Asian | PubMed/MEDLINE | 300 |
| 4: CFIR | PubMed/MEDLINE | 224 |
| 5: Tier 3 — US | PubMed/MEDLINE | 117 |
| 6: Tier 3 — Nordic | PubMed/MEDLINE | 142 |
| **Total retrieved** |  | **2,496** |
| **Duplicates removed** |  | **108 (90 duplicate sets)** |
| **Unique records screened** |  | **2,388** |

Note: Duplicates were identified across all search results collectively.

**4. Screening criteria**

**4.1 Inclusion criteria**

Publications were prioritized for inclusion if they met one or more of the following criteria:

- Reviews and policy analyses providing comprehensive overviews of national community pharmacy systems
- Studies applying CFIR or other implementation science frameworks to pharmacy practice
- Research directly addressing Japan's community pharmacy reforms, including dispensing separation policy, family pharmacist system, Health Support Pharmacy, and medication follow-up services
- Multi-country comparative studies of community pharmacy services or health system performance
- Evaluations of key institutional innovations in community pharmacy (e.g., pharmacist prescribing, clinical service programs, digital health platforms)

**4.2 Exclusion criteria**

Publications were excluded if they met one or more of the following criteria:

- Single-site, small-scale studies with limited generalizability beyond the local context
- Disease-specific pharmacotherapy comparisons without institutional or health system context
- Basic pharmacological or pharmaceutical sciences research
- Studies focused exclusively on hospital pharmacists without relevance to community pharmacy
- Pharmacy education curriculum studies without practice implementation components
- Purely descriptive patient satisfaction surveys without analytical or comparative elements

**4.3 Evidence-driven depth approach**

An evidence-driven depth approach was adopted, whereby the breadth, quality, and relevance of available literature for each country naturally determined the level of analytical detail in the review. Countries with more extensive English-language research literature on community pharmacy implementation (particularly the United Kingdom and Canada) received more detailed treatment than those with less accessible evidence bases.

**5. Screening results and supplementary source identification**

From 2,388 unique records, title and abstract screening was conducted by the lead author (RU). A total of 74 publications were selected through screening based on the predefined inclusion and exclusion criteria described above.

An additional 13 supplementary sources were identified through hand-searching and reference tracking. These comprised:

- Government reports and legislative research documents essential to the analysis of Japan's pharmacy reform trajectory (references [6, 7, 8] in the main text)
- Primary analytical and supplementary framework references (references [1, 2, 14, 16, 20] in the main text)
- Methodological literature supporting the narrative review design (references [17, 18, 19] in the main text)
- A historical analysis of the Japanese pharmacist's role during the COVID-19 pandemic (reference [21] in the main text)
- A reporting guideline adopted to enhance transparency of the literature selection process (reference [22] in the main text)

In total, 87 publications were included in the analysis.

**6. Search period rationale**

The primary search period was January 2016 to February 2026 for Searches 1 (Japan), 2 (Tier 1 countries), and 5 (US MTM). This period was selected to capture the most recent decade of community pharmacy reform literature, beginning from the year following Japan's 2015 "Pharmacy Vision for Patients" policy document.

For Searches 3 (Tier 2 Asian countries), 4 (CFIR), and 6 (Nordic countries), the search period was extended back to January 2011 to capture earlier foundational literature. This extension was necessary because CFIR-pharmacy literature began emerging around 2013, key Asian prescribing–dispensing separation studies were published in the early 2010s, and Nordic pharmacy deregulation and reform literature included important pre-2016 publications.

**7. Limitations of the search strategy**

The following limitations are acknowledged:

- Embase was not searched. While Embase provides broader European pharmacy practice coverage, resource constraints necessitated focusing on PubMed/MEDLINE and Ichushi-Web. This may have resulted in some relevant European publications being missed.
- Grey literature searching was limited to hand-searching and reference tracking of government reports and legislative documents. Systematic grey literature searches of organizational repositories (e.g., WHO, FIP, national pharmacy associations) were not conducted.
- Many Japanese-language sources in Ichushi-Web lacked English abstracts, requiring reliance on title-based assessment for initial screening of these records.
- Screening was conducted by a single reviewer (RU), which may introduce selection bias. However, the narrative review methodology and predefined criteria were designed to mitigate this limitation.

**8. Reporting transparency**

While this study employs a narrative review methodology, a flow diagram adapted from the PRISMA 2020 template [22] is provided as Figure 2 in the main text to enhance transparency of the literature selection process. This flow diagram illustrates the flow of records through identification, screening, and inclusion and is provided for reporting transparency; it does not indicate compliance with systematic or scoping review methodology.
